# The Multitasking System of Swarm Robot based on Null-Space-Behavioral Control Combined with Fuzzy Logic

**DOI:** 10.3390/mi8120357

**Published:** 2017-12-09

**Authors:** Nga Le Thi Thuy, Thang Nguyen Trong

**Affiliations:** 1 Department of Cybernetics, University of Transport and Communication, Hanoi 122000, Vietnam; lethuynga@utc.edu.vn; 2 Department of Electrical Engineering and Automation, Haiphong Private University, Haiphong 181810, Vietnam

**Keywords:** swarm robot, Lyapunov theory, fuzzy control, multitasking

## Abstract

A swarm robot is a collection of large numbers of simple robots used to perform complex tasks that a single robot cannot perform or only perform ineffectively. The swarm robot works successfully only when the cooperation mechanism among individual robots is satisfied. The cooperation mechanism studied in this article ensures the formation and the distance between each pair of individual robots while moving to their destination while avoiding obstacles. The solved problems in this article include; controlling the suction/thrust force between each pair of individual robots in the swarm based on the fuzzy logic structure of the Singer-Input-Singer-Output under Mamdani law; demonstrating the stability of the system based on the Lyapunov theory; and applying control to the multitasking system of the swarm robot based on Null-Space-Behavioral control. Finally, the simulation results make certain that all the individual robots assemble after moving and avoid obstacles.

## 1. Introduction

A flock is a gathering of a group of creatures, found in nature of many different species such as insect pests, ants, bees, termites, fish, etc. Flocking behavior can accomplish tasks that surpass the ability of the individual. So, researchers have modeled flocking activity, inheriting these advantages to apply to multi-robots. By the end of the 1980s, scientists had researched and built robotic teams with the capability of working and coordinating to perform a specific task [1].

The characteristics of the swarm robot are intellectual without requiring the complex manufacturing technology in the robot field [2,3]. The swarm robot can be used in many fields such as search [4,5], cleaning [6], and transportation [7,8]. So, swarm robots are increasingly attracting the interest of scientists around the world. A number of successful researches related to flock robots has brought swarm robots to more and more widespread applications in life, such as multi-robot system main principles [9,10,11,12,13,14,15,16], swarm robotics [17,18,19,20], human-multi-robot interaction [21,22], problem-specific works [23,24,25,26], and autonomous underwater vehicles [19,27].

In order that the robot individuals of the swarm can work together to perform a certain task, the first and most important problem that must be addressed is ensuring a collaborative mechanism among robots. When an individual robot performs a task, it must avoid obstacles, but it is not separated or cannot collide with others [28,29]. So, before researching the specialized applications of the swarm robot, the first problem that needs to be solved is ensuring the swarm and the distance among individuals while moving and avoiding obstacles.

Some researchers have solved the above problem as in references [30,31,32,33]. All of these studies are on the interaction between each pair of individuals and between the individual and the environment. The interactions are expressed by the suction/thrust force among individuals, usually explicit mathematical functions, but there is no convincing explanation. In reality, the working environment of the swarm robot is quite complex, frequently changing, and each robot consists of a collection of many details such as the engine, power circuit, control circuit, etc. so it is difficult to determine an explicit mathematical model, or even impossible to identify.

In order for the model system to get closer to nature, this article proposes a solution which uses fuzzy logic for controlling the suction/thrust force between each pair of individuals in the swarm. The advantage of this solution is that the object can be controlled easily without the requirement of knowing the object mathematical equation. Then, the authors applied this method to control the multitasking system of a swarm robot.

Multitasking in a swarm robot means that each individual robot in the swarm must perform multiple tasks at the same time; research [34] has also introduced two basic mechanisms to solve the multitasking problem as follows:

The first is an arbitration mechanism: the controller only obeys the output commands of the higher-priority behavior. The lower-priority behavior can only be performed if the output of the higher-priority behavior is equal to zero. The drawback of this mechanism is that when the behaviors are not inconsistent with each other they still cannot be done at the same time.

The second is the integration mechanism: The command is created based on a combination of several behaviors. The drawback of this mechanism is that when the behaviors are inconsistent with each other, they cannot be done at the same time.

Thus, the arbitration mechanism (competition) only allows one task at the same time, so this mechanism is rarely used. The integration mechanism allows for combining several tasks to achieve a different mission, but it is difficult to perform conflicting tasks. Research [35] has introduced a new method to solve the above limitations, which is the null space behavioral (NSB) control method. Based on this method, the complex task of the swarm robot can be divided into different basic tasks (behaviors), these tasks are properly combined to achieve the objective mission. The main steps of this method are determining the priority assignment of each basic task, then projection of the lower-priority tasks on the null-space of the higher-priority task.

So, the current authors combine the two best-fit methods to control the swarm robot: the first is using fuzzy logic to control the suction/thrust force among the individual robots. The advantage of this method is that the object can be controlled easily without the requirement of knowing the mathematical equation; the second is using the null-space behavioral control method for the multitasking system of swarm robot. The advantage of this method is that it allows the robot to perform many tasks easily at the same time.

## 2. Building the Function of the Suction/Thrust Ford Based on Fuzzy Logic

Considering a set of N individuals in n-dimensional Euclidean space (n ≤ 3). Assume that each individual is a point and ignore their size and mass, the position of the individual (i) in the swarm is $p^{i}=\left[\begin{array}{c} \begin{array}{c} p_{1}^{i}\\ p_{2}^{i} \end{array}\\ \vdots \\ p_{n}^{i} \end{array}\right]\in R^{n}$. The movement of individual robots in a homogeneous environment will depend on the interaction between the individual and all other individuals. The homogeneous environment is an environment where there is no obstruction, no external disturbance affecting the swarm. If pairs of individuals are far apart, they need to move toward each other by suction force in order to maintain the swarm. Conversely, if pairs of individuals are near together, they need to move away from each other by thrust force to avoid collisions. So, the interplay among individuals in the swarm will depend on the distance between the pairs of individuals. The interaction force between two individuals (i and j) is defined as follows:

$$f=f\left(\|p^{j}-p^{i}\|\right)$$

where: $\left\|p^{j}-p^{i}\right\|$ is the distance between two individuals i and j.

The actual distance between two robots (i and j):

$$\sigma_{s}=\|p^{j}-p^{i}\|=\sqrt{{\left(p_{1}^{j}-p_{1}^{i}\right)}^{2}+{\left(p_{2}^{j}-p_{2}^{i}\right)}^{2}+\cdots +{\left(p_{n}^{j}-p_{n}^{i}\right)}^{2}}$$

Named:

(1) $$g\left(\|p^{j}-p^{i}\|\right)=\frac{f\left(\|p^{j}-p^{i}\|\right)}{\left\|p^{j}-p^{i}\right\|}=\frac{f\left(\sigma_{s}\right)}{\sigma_{s}}=g_{a}\left(\sigma_{s}\right)-g_{r}\left(\sigma_{s}\right)$$

where: g(·) is the suction/thrust force between two individuals (i, j), $g_{a}\left(\cdot \right)$ is the thrust force, $g_{r}\left(\cdot \right)$ is the suction force. $\sigma_{s}^{*}$ ∈ R is the distance between two individuals i and j, where the suction force and thrust between the two individuals are in equilibrium, which means:

$$\left(\sigma_{s}\right)\{\begin{array}{ll} =0 & if\;\;\sigma_{s}=\sigma_{s}^{*}\\ <0 & if\;\;0<\sigma_{s}<\sigma_{s}^{*}\\ >0 & if\;\;0<\sigma_{s}^{*}<\sigma_{s} \end{array}$$

${\tilde{\sigma }}_{s}$ is the error between the real distance and the desired distance:

$${\tilde{\sigma }}_{s}=\sigma_{s}-\sigma_{s}^{*}$$

**Figure 1:**
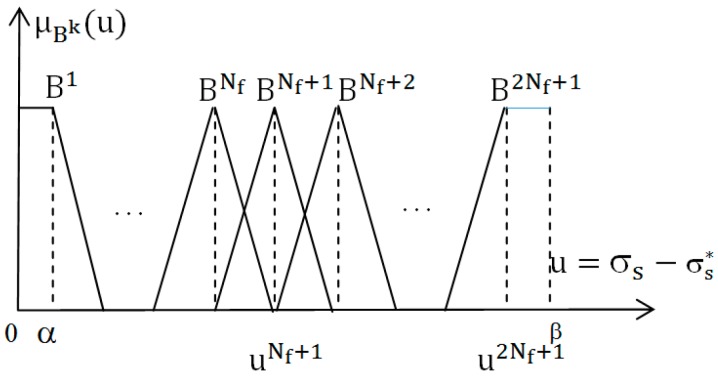
The membership function of the input.

**Figure 2:**
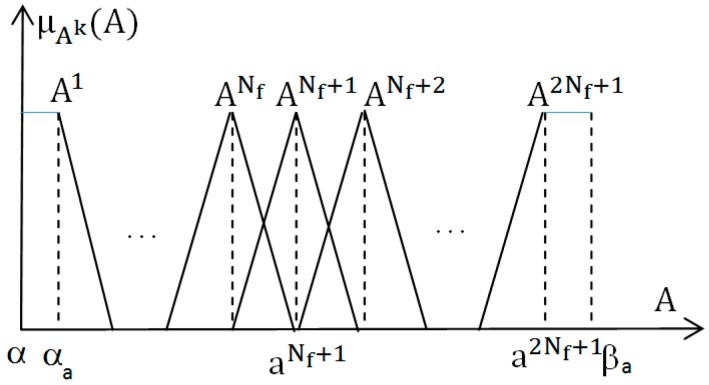
The membership function of the output.

The interaction force f($\sigma_{s}$) among individuals in the swarm is a nonlinear function that depends on the distance between each pair of individuals (i, j). So, we can construct the function $f\left(\sigma_{s}\right)$ based on a Mamdani fuzzy system with the Singer-Input-Singer-Output (SISO) structure as follows:

- First step
  ➢ The input signal is $u={\tilde{\sigma }}_{s}=\sigma_{s}-\sigma_{s}^{*}$, assume that the value domain of u is [α_b_, β_b_] ∈ R, divide this domain into 2Nf + 1 in the range B^k^ as shown in Figure 1.
  ➢ The output signal is A $=f\left(\sigma_{s}-\sigma_{s}^{*}\right)$ with the value domain [α_a_, β_a_], divide this value domain into 2N_f_ + 1 in the range A^k^ as shown in Figure 2 (k = 1, 2, …, 2N_f_ + 1). $a^{k}$ is the focus of the fuzzy range A^k^:
    (2) $$a^{k}\{\begin{array}{ll} <0 & if\;\;k=1,\;2,\ldots N_{f}\;\;\;\;\;\;\;\;\;\;\;\;\;\;\\ =0 & if\;\;k=N_{f}+1\;\;\;\;\;\;\\ >0 & if\;\;k=N_{f}+2,\ldots ,\;2N_{f}+1 \end{array}$$
- The second step: establishing 2Nf + 1 rule IF-THEN with the form: IF: $u=B^{k}$, THEN: $A=A^{k}$
- The third step: defuzzifier using the central area method, we have control laws as follows [36]:
  (3) $$f\left(u\right)=\frac{\sum_{k=1}^{2N_{f}+1}a^{k}\mu_{B^{k}}\left(u\right)}{\sum_{k=1}^{2N_{f}+1}\mu_{B^{k}}\left(u\right)}$$

With the solution to design the fuzzy control through the above three steps, the relationship between the input signal and the output signal is the relationship between the distance and the interaction force between individuals (i, j):

(4) $$\{\begin{array}{l} f\left(\sigma_{s}\right)>0,\;\;if\;\sigma_{s}>\sigma_{s}^{*}\\ f\left(\sigma_{s}\right)<0,\;\;if\;\;0<\sigma_{s}<\sigma_{s}^{*}\\ f\left(\sigma_{s}\right)=0,\;\;if\;\sigma_{s}=\sigma_{s}^{*} \end{array}$$

The fuzzy function $f\left(\sigma_{s}\right)$ is a continuous function which satisfies the following conditions:

- Upper and lower limits:
  (5) $$A_{\min}\leq f\left(\sigma_{s}\right)\leq A_{\max}$$
  where: $A_{\min}=a^{1},{\;\;A}_{\max}=a^{2N_{f}\;+\;1}$
- The equation of a part linearization:
  (6) $$f\left(\sigma_{s}\right)=\frac{\left(a^{k\;+\;1}-a^{k}\right)u+a^{k}u^{k\;+\;1}-a^{k\;+\;1}u^{k}}{u^{k\;+\;1}-u^{k}}$$
  where: $\;u\in \left[u^{k},{\;\;\;u}^{k\;+\;1}\right]$, $k\in \left\{1,\;2,\ldots ,\;2N_{f}\right\}$

$G_{\mathrm{amin}},{\;G}_{\mathrm{amax}}$ is the smallest and largest value of the suction force, $G_{\mathrm{rmin}},{\;G}_{\mathrm{rmax}}$ is the smallest and largest value of the thrust. From (6) we can find the limits of the function $g\left(\sigma_{s}\right)$ as follows:

$$0\leq G_{\mathrm{amin}}\leq g\left(\sigma_{s}\right)\leq G_{\mathrm{amax}},\;\mathrm{if}\;\sigma_{s}>\sigma_{s}^{*}$$

(7) $$-G_{\mathrm{rmin}}\leq g\left(\sigma_{s}\right)\leq G_{\mathrm{rmax}}<0,\;\mathrm{if}\;0<\sigma_{s}<\sigma_{s}^{*}$$

where

$$G_{\mathrm{amax}}=\underset{N_{f}+2\leq k\leq 2N_{f}+1}{\max}\left[\frac{a^{k+1}-a^{k}}{u^{k+1}-u^{k}}\right]$$

$$G_{\mathrm{amin}}=\underset{N_{f}+2\leq k\leq 2N_{f}+1}{\min}\left[\frac{a^{k+1}-a^{k}}{u^{k+1}-u^{k}}\right]$$

$$G_{\mathrm{rmax}}=\underset{1\leq k\leq N_{f}}{\max}\left[\frac{a^{k+1}-a^{k}}{u^{k+1}-u^{k}}\right]$$

$$G_{\mathrm{rmin}}=\underset{1\leq k\leq N_{f}}{\min}\left[\frac{a^{k+1}-a^{k}}{u^{k+1}-u^{k}}\right]$$

## 3. The Stability of the System

Assume that the individuals move in sync and have no delay, all individuals in the swarm know exactly the relative position of all other individuals, the dynamic Equation (1) can be rewritten as follows:

(8) $${\overset{\cdot }{p}}^{i}=\overset{N}{\underset{j=1,j\neq i}{\sum }}f\left(\|p^{j}-p^{i}\|\right)\frac{\left(p^{j}-p^{i}\right)}{\left\|p^{j}-p^{i}\right\|}=\overset{N}{\underset{j=1,j\neq i}{\sum }}g\left(\|p^{j}-p^{i}\|\right)\left(p^{j}-p^{i}\right)$$

where: $\frac{\left(p^{j}-p^{i}\right)}{\left\|p^{j}-p^{i}\right\|}$ displays the direction of the force from the individual (i) to the individual (j), $f\left(\|p^{j}-p^{i}\|\right)$ is the interaction force depending on the distance between the pair of individuals (i, j).

If g(·) > 0, this interaction is the suction force, if g(·) < 0, this interaction is the thrust.

The center of the swarm is defined by the following formula:

(9) $$p^{c}=\frac{1}{N}\overset{N}{\underset{i=1}{\sum }}p^{i}$$

The derivational of the center $p^{c}$:

(10) $${\overset{\cdot }{p}}^{c}=\frac{1}{N}\overset{N}{\underset{i=1}{\sum }}\overset{N}{\underset{j=1,\;j\neq i}{\sum }}g\left(\|p^{j}-p^{i}\|\right)\left(p^{j}-p^{i}\right)\;=\frac{1}{N}\overset{N-1}{\underset{i=1}{\sum }}\overset{N}{\underset{j=i+1}{\sum }}\left[g\left(\|p^{j}-p^{i}\|\right)\left(p^{j}-p^{i}\right)+g\left(\|p^{i}-p^{j}\|\right)\left(p^{i}-p^{j}\right)\right]=0$$

The Equation (10) shows that the center of the swarm robot described by the Equation (8) where the suction/thrust force as Equation (3) is invariant.

The different position between individual (i) and the center is as follows:

$$e^{i}=p^{i}-p^{c}\;(i=1,\;2,\ldots ,N)$$

The derivational of $e^{i}$:

$${\overset{\cdot }{e}}^{i}={\overset{\cdot }{p}}^{i}-{\overset{\cdot }{p}}^{c}={\overset{\cdot }{p}}^{i}$$

Select the Lyapunov function for the individual robot (i):

$$V_{i}=\frac{1}{2}{\left\|e^{i}\right\|}^{2}=\frac{1}{2}e^{\mathrm{iT}}e^{i\;}$$

The derivational of $V_{i}$:

(11) $${\overset{\cdot }{V}}_{i}={\overset{\cdot }{e}}^{\mathrm{iT}}e^{i\;}={\overset{\cdot }{p}}^{\mathrm{iT}}e^{i\;}=\overset{N}{\underset{j=1}{\sum }}g\left(\|p^{j}-p^{i}\|\right){\left(p^{j}-p^{i}\right)}^{T}e^{i}\;$$

The sum of Lyapunov functions of all individuals:

(12) $$V=\overset{N}{\underset{i=1}{\sum }}V_{i}=\frac{1}{2}\overset{N}{\underset{i=1}{\sum }}e^{\mathrm{iT}}e^{i}$$

The derivational of V:

(13) $$\overset{\cdot }{V}=\overset{N}{\underset{i=1}{\sum }}\overset{N}{\underset{j=1}{\sum }}g\left(\|p^{j}-p^{i}\|\right){\left(p^{j}-p^{i}\right)}^{T}e^{i\;}\;=\overset{N-1}{\underset{i=1}{\sum }}\overset{N}{\underset{j=i+1}{\sum }}\left[g\left(\|p^{j}-p^{i}\|\right){\left(p^{j}-p^{i}\right)}^{T}e^{i}+g\left(\|p^{i}-p^{j}\|\right){\left(p^{i}-p^{j}\right)}^{T}e^{j}\right]$$

where: $p^{j}-p^{i}=\left(p^{j}-p^{c}\right)-\left(p^{i}-p^{c}\right)=e^{j}-e^{i}$.

Named: $e=e^{j}-e^{i}$

$$g\left(\|p^{j}-p^{i}\|\right){\left(p^{j}-p^{i}\right)}^{T}e^{i\;}+g\left(\|p^{i}-p^{j}\|\right){\left(p^{i}-p^{j}\right)}^{T}e^{j\;}\;=g\left(\|p^{j}-p^{i}\|\right)\left[{\left(p^{j}-p^{i}\right)}^{T}e^{i\;}+{\left(p^{i}-p^{j}\right)}^{T}e^{j\;}\right]\;=g\left(\|p^{j}-p^{i}\|\right){\left(p^{i}-p^{j}\right)}^{T}\left(e^{j}-e^{i}\right)=-g\left(\|p^{j}-p^{i}\|\right){\left\|p^{j}-p^{i}\right\|}^{2}$$

So:

(14) $$\overset{\cdot }{V}=-\overset{N-1}{\underset{i=1}{\sum }}\overset{N}{\underset{j=i+1}{\sum }}g\left(\|p^{j}-p^{i}\|\right){\left\|p^{j}-p^{i}\right\|}^{2}\;=-\frac{1}{2}\overset{N}{\underset{i=1}{\sum }}\overset{N}{\underset{j=1}{\sum }}g\left(\|p^{j}-p^{i}\|\right){\left\|p^{j}-p^{i}\right\|}^{2}$$

Named: $S_{1}=\left\{\left(i,\;j\right):{\;\|p}^{j}-p^{i}\|>\sigma_{s}^{*}\right\};{\;S}_{2}=\left\{\left(i,\;j\right):{\;\|p}^{j}-p^{i}\|<\sigma_{s}^{*}\right\}$

$$\sum_{S_{1}}=\sum_{i=1}^{N}\sum_{j=1}^{N},\;\;\;\left(i,\;j\right)\in S_{1};\;\sum_{S_{2}}=\sum_{i=1}^{N}\sum_{j=1}^{N},\;\;\;\left(i,\;j\right)\in S_{2}$$

Equation (14) can be rewritten as follows:

(15) $$\overset{\cdot }{V}=-\frac{1}{2}\underset{S_{1}}{\sum }g\left(\|p^{j}-p^{i}\|\right){\left\|p^{j}-p^{i}\right\|}^{2}-\frac{1}{2}\underset{S_{2}}{\sum }g\left(\|p^{j}-p^{i}\|\right){\left\|p^{j}-p^{i}\right\|}^{2}\;=-\frac{1}{2}\left[\underset{S_{1}}{\sum }g\left(\|p^{j}-p^{i}\|\right){\left\|p^{j}-p^{i}\right\|}^{2}+\underset{S_{2}}{\sum }-g\left(\|p^{j}-p^{i}\|\right)\|p^{j}-p^{i}\|{\left\|p^{j}-p^{i}\right\|}^{2}\right]\;-\frac{1}{2}\left[\underset{S_{2}}{\sum }g\left(\|p^{j}-p^{i}\|\right){\left\|p^{j}-p^{i}\right\|}^{2}-\underset{S_{2}}{\sum }-g\left(\|p^{j}-p^{i}\|\right)\|p^{j}-p^{i}\|{\left\|p^{j}-p^{i}\right\|}^{2}\right]\;=-\frac{1}{2}\left[\underset{S_{1}}{\sum }g\left(\|p^{j}-p^{i}\|\right){\left\|p^{j}-p^{i}\right\|}^{2}+\underset{S_{2}}{\sum }-f\left(\|p^{j}-p^{i}\|\right){\left\|p^{j}-p^{i}\right\|}^{2}\right]\;-\frac{1}{2}\left[\underset{S_{2}}{\sum }g\left(\|p^{j}-p^{i}\|\right){\left\|p^{j}-p^{i}\right\|}^{2}+\underset{S_{2}}{\sum }f\left(\|p^{j}-p^{i}\|\right){\left\|p^{j}-p^{i}\right\|}^{2}\right]$$

From Condition (5), we infer:

$$\underset{S_{2}}{\sum }-f\left(\|p^{j}-p^{i}\|\right){\left\|p^{j}-p^{i}\right\|}^{2}\leq A_{\min}\underset{S_{2}}{\sum }{\left\|p^{j}-p^{i}\right\|}^{2};\;\underset{S_{1}}{\sum }g\left(\|p^{j}-p^{i}\|\right){\left\|p^{j}-p^{i}\right\|}^{2}\geq G_{\mathrm{amin}}\underset{S_{1}}{\sum }{\left\|p^{j}-p^{i}\right\|}^{2}$$

Considering the second component of Equation (15):

(16) $$\underset{S_{2}}{\sum }g\left(\|p^{j}-p^{i}\|\right){\left\|p^{j}-p^{i}\right\|}^{2}-\underset{S_{2}}{\sum }-f\left(\|p^{j}-p^{i}\|\right){\left\|p^{j}-p^{i}\right\|}^{2}\;\geq \underset{S_{2}}{\sum }g\left(\|p^{j}-p^{i}\|\right){\left\|p^{j}-p^{i}\right\|}^{2}-\underset{S_{2}}{\sum }A_{\min}{\left\|p^{j}-p^{i}\right\|}^{2}$$

The left side of the Inequality (16):

$$\underset{S_{2}}{\sum }g\left(\|p^{j}-p^{i}\|\right){\left\|p^{j}-p^{i}\right\|}^{2}-\underset{S_{2}}{\sum }A_{\min}{\left\|p^{j}-p^{i}\right\|}^{2}\;=\underset{S_{2}}{\sum }\frac{f\left(\|p^{j}-p^{i}\|\right)-\underline{A}\|p^{j}-p^{i}\|}{\left\|p^{j}-p^{i}\right\|}{\left\|p^{j}-p^{i}\right\|}^{2}$$

Named: $f_{S_{2}}=-f\left(\|p^{j}-p^{i}\|\right)+A_{\min}\|p^{j}-p^{i}\|$

**Figure 3:**
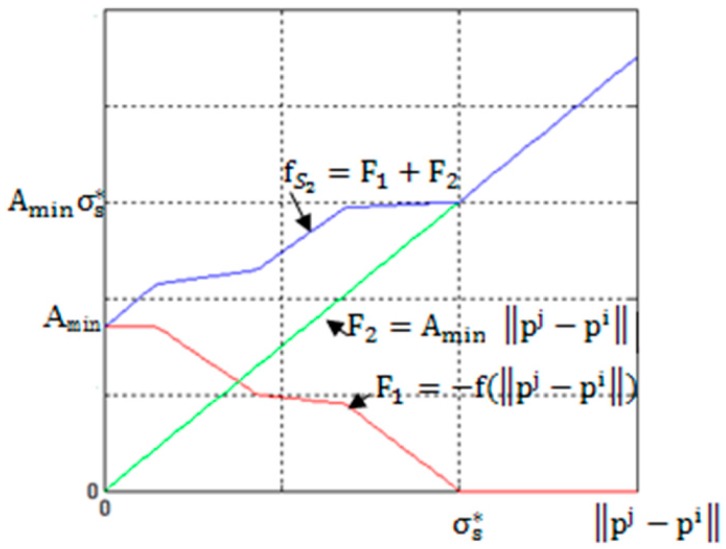
The graph of total $f_{S_{2}}$.

Set β is the largest value of $f_{S_{2}}$ in the domain S_2_. The graph of total $f_{S_{2}}$ is shown in Figure 3.

Named $\beta =A_{\min}\sigma_{s}^{*}$. So:

$$-\underset{S_{2}}{\sum }\frac{-f\left(\|p^{j}-p^{i}\|\right)+\underline{A}\|p^{j}-p^{i}\|}{\left\|p^{j}-p^{i}\right\|}{\left\|p^{j}-p^{i}\right\|}^{2}\leq \beta \underset{S_{2}}{\sum }\|p^{j}-p^{i}\|$$

Thus, the Inequality (16) is equivalent to:

(17) $$\underset{S_{2}}{\sum }g\left(\|p^{j}-p^{i}\|\right){\left\|p^{j}-p^{i}\right\|}^{2}-\underset{S_{2}}{\sum }-f\left(\|p^{j}-p^{i}\|\right){\left\|p^{j}-p^{i}\right\|}^{2}\geq -A_{\min}\sigma_{s}^{*}\underset{S_{2}}{\sum }\|p^{j}-p^{i}\|$$

The first component of Equation (15):

$$\underset{S_{1}}{\sum }g\left(\|p^{j}-p^{i}\|\right){\left\|p^{j}-p^{i}\right\|}^{2}+\underset{S_{2}}{\sum }-f\left(\|p^{j}-p^{i}\|\right){\left\|p^{j}-p^{i}\right\|}^{2}\;=\underset{S_{1}}{\sum }g\left(\|p^{j}-p^{i}\|\right){\left\|p^{j}-p^{i}\right\|}^{2}+\underset{S_{2}}{\sum }A_{\min}{\left\|p^{j}-p^{i}\right\|}^{2}$$

Named $\alpha =\min\left\{G_{\mathrm{amin}},{\;A}_{\min}\right\}$, we have:

(18) $$\underset{S_{1}}{\sum }g\left(\|p^{j}-p^{i}\|\right){\left\|p^{j}-p^{i}\right\|}^{2}+\underset{S_{2}}{\sum }-f\left(\|p^{j}-p^{i}\|\right){\left\|p^{j}-p^{i}\right\|}^{2}\geq \alpha \underset{S_{1}\cup^{\text{​}}S_{2}}{\sum }{\left\|p^{j}-p^{i}\right\|}^{2}$$

Combination of Inequalities (16) and (18), with $\|p^{j}-p^{i}\|\leq \sigma_{s}^{*},\;\left(i,\;j\right)\in S_{2}$, we have:

(19) $$\overset{\cdot }{V}\leq -\alpha \overset{N}{\underset{i=1}{\sum }}\overset{N}{\underset{j=1}{\sum }}{\left\|p^{j}-p^{i}\right\|}^{2}+{\;A}_{\min}{\sigma_{s}^{*}}^{2}$$

From the definition of the swarm robot center, we have:

(20) $$\overset{N}{\underset{j=1}{\sum }}p^{j}={\mathrm{Np}}^{c}$$

Two sides of (20) minus ${\mathrm{Ne}}^{i}$, we have:

$$\overset{N}{\underset{j=1}{\sum }}\left(p^{i}-p^{j}\right)=N\left(p^{i}-p^{c}\right)$$

Thus, the sum of the squared deviations is given by the formula:

(21) $$\overset{N}{\underset{i=1}{\sum }}{\left\|e^{i}\right\|}^{2}=\frac{1}{N}\overset{N}{\underset{i=1}{\sum }}\overset{N}{\underset{j=1}{\sum }}{\left(p^{i}-p^{j}\right)}^{T}e^{i}=\frac{1}{N}\overset{N-1}{\underset{i=1}{\sum }}\overset{N}{\underset{j=1}{\sum }}{\left\|p^{j}-p^{i}\right\|}^{2}\;=\frac{1}{2N}\overset{N}{\underset{i=1}{\sum }}\overset{N}{\underset{j=1}{\sum }}{\left\|p^{j}-p^{i}\right\|}^{2}$$

Combining Equations (17) and (21) we have:

$$\overset{\cdot }{V}\leq -2N\alpha \overset{N}{\underset{i=1}{\sum }}{\left\|e^{i}\right\|}^{2}+{\;A}_{\min}{\sigma_{s}^{*}}^{2}$$

$\overset{\cdot }{V}<0$ when $\sum_{i=1}^{N}{\left\|e^{i}\right\|}^{2}>\frac{A_{\min}{\sigma_{s}^{*}}^{2}}{2N\alpha }$. So, we can conclude with the following theorem:

**Theorem:**  *Swarm robots are modeled by Equation (8) with the fuzzy control law of suction/thrust force constructed according to (3), satisfying Conditions (4). After a period, all individuals of the swarm will be converged in a restricted area by:*

(22) $${Ω}_{\sigma }=\left\{\sum {\left\|p^{j}-p^{i}\right\|}^{2}\leq \sigma^{2}\right\}$$

where $\sigma =\sqrt{\frac{A_{\min}{\sigma_{s}^{*}}^{2}}{2N\alpha }}=\alpha_{s}^{*}\sqrt{\frac{A_{\min}}{2N\alpha }}$ is the convergent radius of the swarm.

The effect of the parameters on the restricted area of the swarm $\left({Ω}_{\sigma }\right)$ is as follows:

- If $A_{\min}$ increases, the thrust increases, the restricted area of the swarm robot increases.
- If α increases, the restricted area of the swarm robot decreases.
- If the size of the swarm (N) is bigger, the restricted area is lower.

## 4. Multitasking-Control System of Swarm Robot

When the robots perform the task of moving to a destination, on the way they must avoid obstacles. So, each robot in the swarm has three tasks as follows:

- The first task is avoiding obstacles
- The second task is moving to the destination.
- The third task is maintaining the swarm: Avoiding collisions among individuals in the swarm, but not splitting the group.

**Figure 4:**
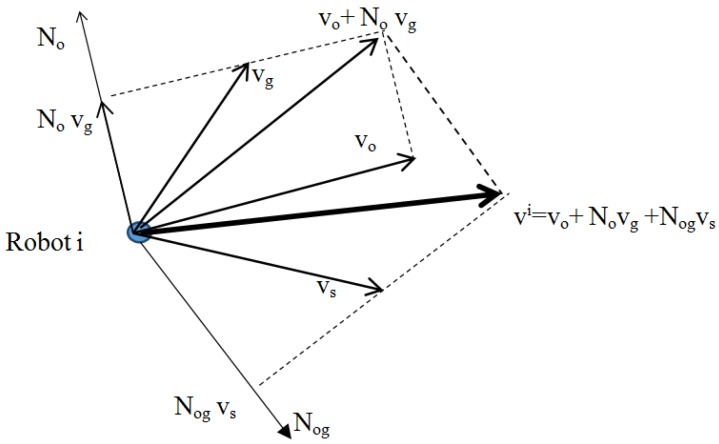
The speed vector of each robot based on the null-space behavioral control technique.

In order for the robot to perform the above tasks, the supervisor selects the priority of the tasks. In this study, the priority of the tasks in order is: Avoiding obstacles, moving to the destination, and maintaining the swarm. Assume that the obstacles are static and are known then the speed vector of each robot based on the null- space behavioral control technique [36] is calculated according to Figure 4.

The speed of the robot (i) is determined as follows:

$$v^{i}=v_{o}+N_{o}v_{g}+N_{\mathrm{og}}v_{s}$$

where $v_{o}$, $v_{g}$, $v_{s}$ are the speed vectors performing the tasks: Avoiding obstacles, moving to the destination and maintaining the swarm. $N_{o}$, $N_{\mathrm{og}}$ are the projection matrixes according to the priority of the tasks.

### 4.1. Determining the Speed Component Avoiding Obstacles

Assume that in the working environment of the swarm the robot has M obstructions, $p^{o_{m}}\;=\left[\begin{array}{c} p_{1}^{o_{m}}\\ p_{2}^{o_{m}}\\ \vdots \\ p_{n}^{o_{m}} \end{array}\right]\in R^{n\times 1}$ is the position of the obstruction (m) in n-dimensional space, (m = 1 ÷ M).

σ_o_ ∈ R is the actual distance between the individual robot (i) and the obstacle (m):

$$\sigma_{o}=\|p^{o_{m}}-p^{i}\|=\sqrt{{\left(p_{1}^{o_{m}}-p_{1}^{i}\right)}^{2}+{\left(p_{2}^{o_{m}}-p_{2}^{i}\right)}^{2}+\cdots +{\left(p_{n}^{o_{m}}-p_{n}^{i}\right)}^{2}}$$

The purpose of the speed component avoiding obstacle is that if the obstacle lies in the moving way, the robot must be kept away from the obstacle at a safe distance $\sigma_{o,d}=\sigma_{o}^{*}$, if the obstacle is outside the safe area of the robot, the obstacle does not affect the movement speed of the robot. So, the movement speed of the robot depends on the distance between the robot and the obstacle.

The Jacobi Matrix $J_{o}\in R^{M\times n}$ shows the movement speed of the robot avoiding obstacles:

(23) $$J_{o}=\left[\begin{array}{c} {\left[\frac{p^{o_{1}}-p^{i}}{\left\|p^{o_{1}}-p^{i}\right\|}\right]}^{T}\\ {\left[\frac{p^{o_{2}}-p^{i}}{\left\|p^{o_{2}}-p^{i}\right\|}\right]}^{T}\\ \vdots \\ {\left[\frac{p^{o_{M}}-p^{i}}{\left\|p^{o_{M}}-p^{i}\right\|}\right]}^{T} \end{array}\right]={\hat{p}}_{\mathrm{io}}^{T}$$

The Matrix inverse of J_o_:

$$J_{o}^{+}={\hat{p}}_{\mathrm{io}},\;J_{o}^{+}\in R^{n\times M}$$

The projection matrix of J_o_:

(24) $$N_{o}=I_{n}-{\hat{p}}_{\mathrm{io}}{\hat{p}}_{\mathrm{io}}^{T},\;N_{o}\in R^{n\;\times \;n}$$

where I_n_ is the unit matrix.

The speed component avoiding the obstacle is defined as follows:

(25) $$v_{o}=-k_{\mathrm{vo}}J_{o}^{+}\left(\sigma_{o}-\sigma_{o}^{*}\right)=-k_{\mathrm{vo}}J_{o}^{+}{\tilde{\sigma }}_{o}$$

where $k_{\mathrm{vo}}$ is a negative coefficient, ${\tilde{\sigma }}_{o}=\sigma_{o}-\sigma_{o}^{*}$ is the error between the actual distance and the desired distance from the robot to the obstacle.

### 4.2. Determining the Speed Component Moving to the Target

Named $p^{g}\;=\left[\begin{array}{c} p_{1}^{g}\\ p_{2}^{g}\\ \vdots \\ p_{n}^{g} \end{array}\right]\in R^{n\times 1}$ is the position of the target, σ_g_∈ R is the actual distance between the robot (i) and the target, $\sigma_{g}$ is calculated according to the formula:

$$\sigma_{g}=\|p^{g}-p^{i}\|=\sqrt{{\left(p_{1}^{g}-p_{1}^{i}\right)}^{2}+{\left(p_{2}^{g}-p_{2}^{i}\right)}^{2}+\cdots +{\left(p_{n}^{g}-p_{n}^{i}\right)}^{2}}$$

The purpose of the speed component moving to the target is that the desired distance ($\sigma_{g}^{*})$ is equal to 0:

$$\sigma_{g,d}=\sigma_{g}^{*}=0$$

The Jacobi Matrix $J_{g}\in R^{1\times n}$:

(26) $$J_{g}={\left[\frac{p^{g}-p^{i}}{\left\|p^{g}-p^{i}\right\|}\right]}^{T}={\hat{p}}_{\mathrm{ig}}^{T}$$

The Matrix inverse of J_g_:

$$J_{g}^{+}={\hat{p}}_{\mathrm{ig}},{\;\;\;J}_{g}^{+}\in R^{n\times 1}$$

The projection matrix of J_g_:

(27) $$N_{g}=I_{n}-{\hat{p}}_{\mathrm{ig}}{\hat{p}}_{\mathrm{ig}}^{T},\;N_{g}\in R^{n\times n}$$

The speed component moving to the target (i) is rewritten as follows:

(28) $$v_{g}=k_{\mathrm{vg}}J_{g}^{+}\left(\sigma_{g}-\sigma_{g}^{*}\right)=k_{\mathrm{vg}}J_{g}^{+}{\tilde{\sigma }}_{g}$$

where $k_{\mathrm{vg}}$ is a positive coefficient, ${\tilde{\sigma }}_{g}=\sigma_{g}-\sigma_{g}^{*}$ is the error between the actual distance and the desired distance from the robot to the target:

$${\tilde{\sigma }}_{g}=\sigma_{g}-\sigma_{g}^{*}=\sigma_{g}$$

### 4.3. Determining the Maintained-Swarm Speed Component

The purpose of the maintained-swarm speed component is keeping $\sigma_{s}=\sigma_{s}^{*}\in R$. In this condition, the difference between the actual distance and the desired distance is:

$${\tilde{\sigma }}_{s}=\sigma_{s}-\sigma_{s}^{*}$$

From the model Equation (8) of the individual robot (i), the Jacobi matrix $J_{s}$:

(29) $$J_{s}={\hat{p}}_{s}^{T}=\left[\begin{array}{c} J_{s1}\\ J_{s2}\\ \vdots \\ J_{\mathrm{sN}} \end{array}\right]=\left[\begin{array}{c} {\hat{p}}_{s1}^{T}\\ {\hat{p}}_{s2}^{T}\\ \vdots \\ {\hat{p}}_{\mathrm{sN}}^{T} \end{array}\right]=\left[\begin{array}{c} {\left[\frac{p^{1}-p^{i}}{\left\|p^{1}-p^{i}\right\|}\right]}^{T}\\ {\left[\frac{p^{2}-p^{i}}{\left\|p^{2}-p^{i}\right\|}\right]}^{T}\\ \vdots \\ {\left[\frac{p^{N}-p^{i}}{\left\|p^{N}-p^{i}\right\|}\right]}^{T} \end{array}\right]\in R^{N\times n}$$

The Matrix inverse of J_s_:

(30) $$J_{s}^{+}={\hat{p}}_{s}={\left[\begin{array}{c} J_{s1}\\ J_{s2}\\ \vdots \\ J_{\mathrm{sN}} \end{array}\right]}^{T}={\left[\begin{array}{c} {\hat{p}}_{s1}^{T}\\ {\hat{p}}_{s2}^{T}\\ \vdots \\ {\hat{p}}_{\mathrm{sN}}^{T} \end{array}\right]}^{T}={\left[\begin{array}{c} {\left[\frac{p^{1}-p^{i}}{\left\|p^{1}-p^{i}\right\|}\right]}^{T}\\ {\left[\frac{p^{2}-p^{i}}{\left\|p^{2}-p^{i}\right\|}\right]}^{T}\\ \vdots \\ {\left[\frac{p^{N}-p^{i}}{\left\|p^{N}-p^{i}\right\|}\right]}^{T} \end{array}\right]}^{T}\in R^{n\times N}$$

The projection matrix of J_s_:

(31) $$N_{s}=I_{n}-{\hat{p}}_{s}{\hat{p}}_{s}^{T},\;N_{s}\in R^{n\times n}$$

The maintained-swarm speed component of the individual robot (i) is defined as follows:

(32) $$v_{s}=J_{s}^{+}f\left({\tilde{\sigma }}_{s}\right)\in R^{n\times 1}$$

Combining all the speed vectors of each robot when performing all three tasks based on the NSB method is shown in Figure 4:

(33) $$v^{i}=v_{o}+N_{o}v_{g}+N_{\mathrm{og}}v_{s}=-k_{\mathrm{vo}}J_{o}^{+}{\tilde{\sigma }}_{o}+k_{\mathrm{vg}}N_{o}J_{g}^{+}{\tilde{\sigma }}_{g}+N_{\mathrm{og}}J_{s}^{+}f\left({\tilde{\sigma }}_{s}\right)$$

where: $v^{i}\in R^{n\times 1},{\;J}_{\mathrm{og}}=\left[\begin{array}{c} J_{o}\\ J_{g} \end{array}\right],{\;\;J}_{\mathrm{og}}\in R^{\left(M+1\right)\times n}$, $N_{\mathrm{og}}=I_{n}-J_{\mathrm{og}}^{+}J_{\mathrm{og}},{\;\;\;N}_{\mathrm{og}}\in R^{n\times n}$.

### 4.4. The Algorithm of Swarm Robot Control for Performing Multiple-Task

The algorithm of swarm robot control for performing multiple-tasks includes the following steps:

- The first step
  ➢ Enter the number (N) of robots in the swarm.
  ➢ Enter the number (M) of obstacles in the moving space.
  ➢ Initially the position of individual robots in n-dimensional space:
    $$p^{1}=\left[\begin{array}{c} \begin{array}{c} p_{1}^{1}\\ p_{2}^{1} \end{array}\\ \vdots \\ p_{n}^{1} \end{array}\right],\;p^{2}=\left[\begin{array}{c} \begin{array}{c} p_{1}^{2}\\ p_{2}^{2} \end{array}\\ \vdots \\ p_{n}^{2} \end{array}\right],\;\ldots \;p^{N}=\left[\begin{array}{c} \begin{array}{c} p_{1}^{N}\\ p_{2}^{N} \end{array}\\ \vdots \\ p_{n}^{N} \end{array}\right]$$
  ➢ Placement of M obstacles and the destination (g) in n-dimensional space:
    $$p^{o1}=\left[\begin{array}{c} \begin{array}{c} p_{1}^{o1}\\ p_{2}^{o1} \end{array}\\ \vdots \\ p_{n}^{o1} \end{array}\right],\;p^{o2}=\left[\begin{array}{c} \begin{array}{c} p_{1}^{o2}\\ p_{2}^{o2} \end{array}\\ \vdots \\ p_{n}^{o2} \end{array}\right],\;\ldots p^{\mathrm{oM}}=\left[\begin{array}{c} \begin{array}{c} p_{1}^{\mathrm{oM}}\\ p_{2}^{\mathrm{oM}} \end{array}\\ \vdots \\ p_{n}^{\mathrm{oM}} \end{array}\right],\;p^{g}=\left[\begin{array}{c} \begin{array}{c} p_{1}^{g}\\ p_{2}^{g} \end{array}\\ \vdots \\ p_{n}^{g} \end{array}\right]$$
  ➢ Enter the safe distance between the individual robot and obstacle $\sigma_{o}^{*}$, the safe distance among robot individuals $\sigma_{s}^{*}$
  ➢ Enter the coefficients $k_{\mathrm{vo}}$ and $k_{\mathrm{vg}}$
  ➢ Enter the number of steps to calculate (K).
- The second step
  ➢ Calculating the distance between each robot (i) and each obstacle $\sigma_{o}$, the distance between each robot and target, the distance between robot (i) and robot (j).
  ➢ Calculating the suction/thrust force $f\left(\sigma_{s}\right)$ according to Equation (3), satisfying Condition (4).
- The third step
  ➢ Comparing the actual distance and safe distance from the robot (i) to the obstacle (m):
    ▪ If $\sigma_{o}\geq \sigma_{o}^{*}$, the robot (i) does not need to avoid the obstacle (o), it means ${\;J}_{o}=\left[0\right]$.
    ▪ If $\sigma_{o}<\sigma_{o}^{*}$, the robot (i) needs to avoid the obstacle (o), calculating ${\;J}_{o}$ by Formula (26). Calculating $J_{o}^{+}$, ${\;N}_{o}$, ${\;v}_{o}$.
  ➢ Comparing the actual distance and the desired distance from the robot (i) to the target:
    ▪ If $\sigma_{g}=0$, the robot (i) reached the target g, $J_{g}=\left[0\right]$.
    ▪ If $\sigma_{g}>0$, the robot (i) has not reached the target, calculating ${\;J}_{g}$ by the Formula (24).
    ▪ Calculating $J_{g}^{+}$,${\;N}_{g}$,${\;v}_{g}$, calculating: $J_{\mathrm{og}}$, $J_{\mathrm{og}}^{+}$, $N_{\mathrm{og}}$.
  ➢ Comparing the actual distance and the desired distance from the robot (i) to the robot (j):
    ▪ If $\sigma_{s}>\sigma_{s}^{*}$, the robot (i) and the robot (j) move towards each other by the suction force $f\left(\sigma_{s}\right)>0$.
    ▪ If $\sigma_{s}<\sigma_{s}^{*}$, the robot (i) and the robot (j) move away from each other by the thrust force $\;f\left(\sigma_{s}\right)<0$.
    If $\sigma_{s}=\sigma_{s}^{*}$, the robot (i) and the robot (j) keep their route because of $f\left(\sigma_{s_{i}}\right)=0$.
    Calculating $J_{s}$, $J_{s}^{+}$, $v_{s}$.
- The fourth step
  ➢ The speed of the individual (i) at the step k (k = 0 ÷ K − 1) is determined by the formula:
    $$v^{i}\left[k\right]=v_{o}\left[k\right]+N_{o}\left[k\right]v_{g}\left[k\right]+N_{\mathrm{og}}\left[k\right]v_{s}\left[k\right]$$
  ➢ The distance moved of the robot (i) in a step time(Δt):
    $$\Delta S^{i}\left[k+1\right]=\Delta S^{i}\left[k\right]+v^{i}\left[k\right]\;\times \;\Delta t$$
  ➢ The new position of the robot (i) after k + 1 steps:
    $$p^{i}\left[k+1\right]=p^{i}\left[k\right]+\Delta S^{i}\left[k+1\right]$$

Repeat from the second step to the fourth step until all individuals converge at the target and after K calculation steps.

## 5. Simulation Results and Analysis

**Figure 5:**
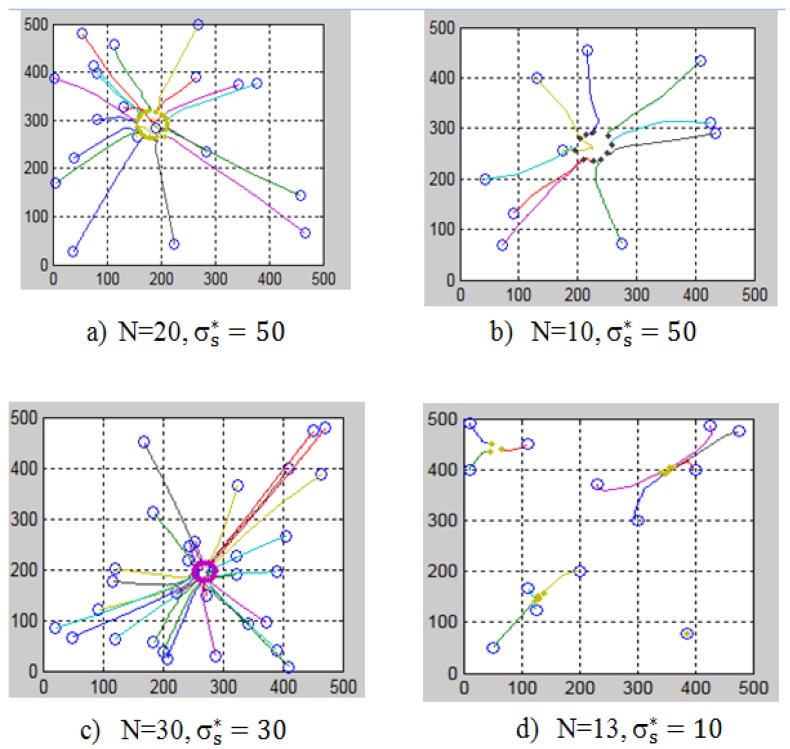
The convergence process of the swarm robot without obstacles.

We ran the simulation in the two-dimensional coordinate system [500, 500]. The initial position of the robots, obstacles, and targets are random. The convergence process of the swarm robot without obstacles is shown in Figure 5.

R is the actual convergence radius of the swarm robot, R is also the distance from the nearest robot to the center of the swarm. The results of the calculation of the parameters in certain cases are presented in Table 1.

The results in Table 1 show that:

- If the size of the swarm (N) increases, the convergence radius decreases;
- If the safe distance $\left(\sigma_{s}^{*}\right)$ increases, the convergence radius increases;
- The actual convergent radius (R) is always smaller than the calculated value (σ).

The simulation results are consistent with the theorem that the authors expressed above.

**Figure 6:**
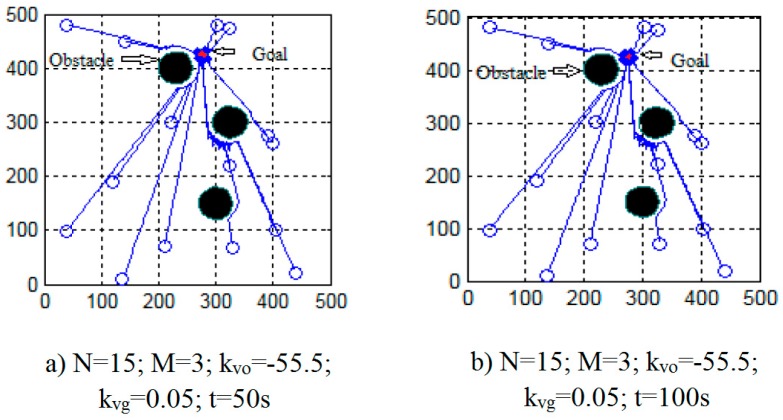
The simulation results of the multi task process after a period.

**Figure 7:**
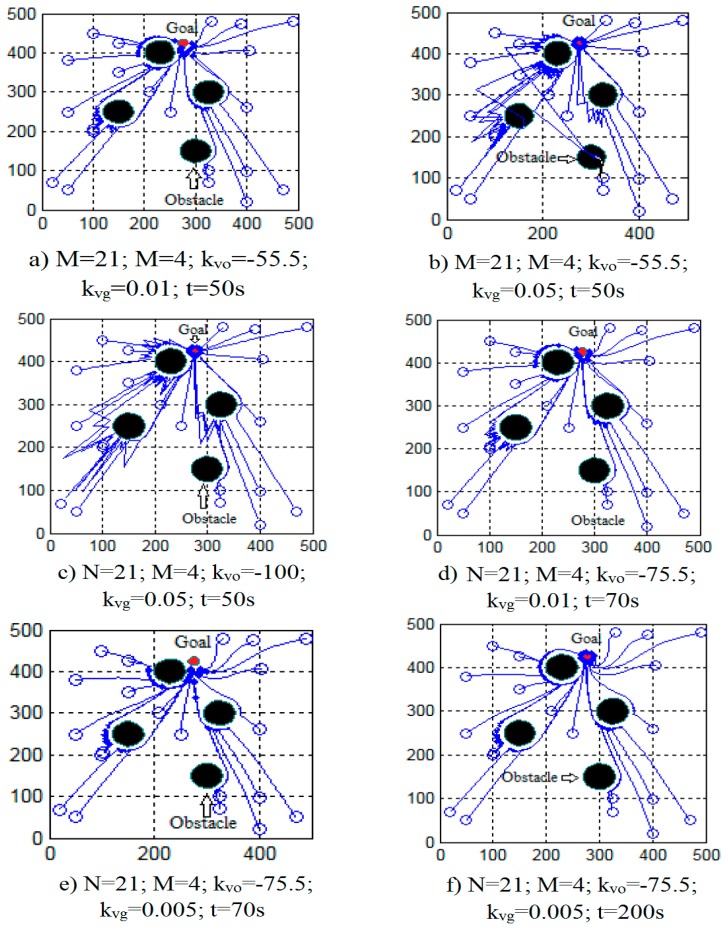
The simulation results of the multitask process when the coefficients $k_{\mathrm{vo}\;}$ and $k_{\mathrm{vg}}$ are changing.

Run the swarm robot model with the obstacles, then the simulation results when the number of robots are changing and the priority coefficient is changing are shown in Figure 6 and Figure 7.

Figure 6 shows that all individual robots have moved to their destination and avoided obstacles along the way. After individual robots converge to the destination, they only move around the destination area and do not move away.

The simulation results when changing the priority coefficients $k_{\mathrm{vo}}$ and $k_{\mathrm{vg}}$ are shown in Figure 7. From the simulation results, we see:

- If the coefficient $k_{\mathrm{vg}}$ is larger, the individual movement to the target is faster.
- If we want to increase the coefficient $k_{\mathrm{vg}}$ but not let the robot collide with obstacles, we must reduce the coefficient $k_{\mathrm{vo}}$. This means, if $k_{\mathrm{vg}}$ is more positive, $k_{\mathrm{vo}}$ must be more negative.
- If the number of obstacles (M) is bigger, the avoid-obstacle-coefficient ($k_{\mathrm{vo}})$ must be more negative. If the coefficient $k_{\mathrm{vo}}$ is more negative, the ability of the robots to avoid obstacles is better, but the moving time to the destination will be longer.

## 6. Conclusions

In this paper, the authors applied both fuzzy logic to control the suction/thrust force between individuals in the swarm, and the null-space behavioral control technique to determine the total speed vector of each robot. The proposed solution is close to nature with high flexibility by selecting the input/output, defuzzifier, and the fuzzy rules. The simulation results coincide with the theories proposed by the authors. Compared with the previous methods, the advantage of our proposed method is that the object can be controlled easily without the requirement of knowing the mathematical equation. We controlled effectively a swarm robot without the object model equations. All individuals of the swarm move together to the destination without collision, and ensure the task of avoiding obstacles.

## Figures and Tables

**Table 1:** The parameters in certain cases.

N	$\sigma_{s}^{*}$	α	β	σ	R
10	10	0.02	9.74	15.61	6.18
10	30	0.02	25.37	43.62	17.06
20	10	0.02	8.40	10.25	4.10
20	30	0.02	25.33	30.82	15.18
30	10	0.02	8.23	8.77	3.91
30	30	0.02	25.25	25.23	15.04
